# Biomass addition alters community assembly in ultrafiltration membrane biofilms

**DOI:** 10.1038/s41598-020-68460-x

**Published:** 2020-07-14

**Authors:** Marisa O. D. Silva, Jakob Pernthaler

**Affiliations:** Limnological Station, Department of Plant and Microbial Biology, University of Zurich, Seestrasse 187, 8802 Kilchberg, Switzerland

**Keywords:** Microbiome, Microbial communities, Water microbiology, Applied microbiology, Biofilms

## Abstract

Freshwater biofilms assemble from a pool of rare water column genotypes. Random density fluctuations and temporal species turnover of functionally equivalent potential colonizers result in compositional variability of newly formed biofilm communities. We hypothesized that stronger environmental filtering as induced by enhanced substrate levels might reduce the impact of a locally variable pool of colonizers and instead select for more universal habitat specialists. Our model were heterotrophic biofilms that form on membranes during gravity-driven ultrafiltration of lake water. In four separate experiments, biomass of the cyanobacterium *Microcystis* was added to the feed water of one set of treatments (BM) and the resulting biofilm communities were compared to unamended controls (CTRL). Biomass addition led to a significant shift of community assembly processes: Replicate BM biofilms were more similar to each other than by chance in 3 of 4 experiments, whereas the opposite was the case for CTRL communities. Moreover, BM communities were more stochastically assembled across experiments from a common ‘regional’ pool of biofilm colonizers, whereas the composition of CTRL communities was mainly determined by experiment-specific ‘local’ genotypes. Interestingly, community assembly processes were also related to both, physiology (aerobic vs. anaerobic lifestyle) and the phylogenetic affiliation of biofilm bacteria.

## Introduction

Virtually every submerged natural and artificial surface attracts the spontaneous formation of biofilms^1^. This biofouling process has important ecological, hygienic and economic consequences^2,3^. Many if not most microbes in lakes and streams are capable of surface attachment, as reflected by the high diversity in the various types of freshwater biofilms^4–8^. Biofilm-forming bacteria have a dual life style and undergo profound physiological transitions between the planktonic and sessile phase^9^. However, there are differences in the respective affinity of lacustrine bacteria to the attached life style, and some abundant genera are even exclusively planktonic^10,11^. As a consequence, the free-living and biofilm-associated freshwater microbial assemblages substantially differ in composition^12,13^. Population dynamics and community structure in multispecies biofilms are strongly shaped by interspecific interactions^14^, and environmental filtering has been proposed as a major assembly mechanism in, e.g., stream biofilm^4^.

The relationship between the community composition of biofilms with that of the source assemblage is poorly understood. With the possible exception of highly productive systems such as wastewater^15^, the dominant genotypes of aquatic biofilms appear to be rare in the surrounding water. These infrequent biofilm forming bacteria often have a ‘tychoplanktic’ life style in that they originate from a variety of non-pelagic habitats, such as suspended organic aggregates^16^, metazoan guts^6^ or body surfaces^17^, epiphytic or epilithic biofilms^7^, or terrestrial influx^18^. Community composition within these biofilm types may spatially vary or change over time^13,19^, thereby leading to an ever-shifting pool of potential colonizers of novel surfaces. These mechanisms add an element of chance to the basically niche-driven^4^ selection of biofilm bacteria: For one, the functional redundancy of co-existing taxa in combination with priority effects^20^ will instigate a ‘competitive lottery’ of potential colonizers from the ‘local’ species pool^21^, i.e. those species that are present in the individual source communities. Secondly, larger scale spatial heterogeneity^22^ or temporal (e.g., seasonal) species turnover^23^ in the water column will create additional variation at the level of the ‘regional’ pool, defined as the totality of species in all source communities.

While the conditions in biofilms always differ from that of the surrounding water phase^12^, their growth on inert surfaces such as rocks, glass slides, sand grains or porous membranes arguably depends on external substrates such as dissolved and particulate organic matter^24^. It is conceivable that the quantity or quality of externally provided substrates may also shift the respective importance of the ‘local’ and ‘regional’ pools of potential colonizers during biofilm community assembly, e.g., by affecting functional redundancy and the balance between competitors in the source assemblage^25^. We addressed this hypothesis using biofilms in experimental devices designed to investigate the potential of gravity-driven membrane filtration (GDM) for decentralized drinking water production^26,27^. We synoptically analysed microbial community composition and assembly processes in four independent experiments that were performed over the course of several years^27–29^ with feed water from a single lake.

## Results

### Diversity and composition of biofilm communities

The complete sequencing dataset comprised 3,495 OTUs and approximately 10^6^ reads. It was rarefied to the sample with the lowest read number (1.34 × 10^4^) by random read exclusion^27^. The normalized data set used for all subsequent analyses consisted of 2,721 OTUs with a total of 0.21 × 10^6^ reads. The CTRL treatment had significantly higher OTU richness (734 ± 165, mean ± standard deviation) than the BM treatment (357 ± 63) (Student’s *t* test, n = 8, *p* < 0.001). The two treatments shared 786 genotypes that together formed 87% of total reads. Sixteen OTUs, representing almost one quarter of total reads, were present in every single community (Table 1).

**Table 1:** OTUs found in all biological replicates of all treatments and experiments.

Taxonomy	Accession number (% identity with closest known sequence)	Proportion of total reads (%)	BM treatment: % of reads	CTRL treatment: % of reads	Contribution (%)
*Rhodoferax* sp.	LN870966 (100)	4.6	4.2	0.4	21.28**
*Paucibacter* sp.	KM187599 (99.6)	3.9	3.8	0.1	18.41***
*Pseudoxanthomonas* sp.	LN560679 (100)	2.9	2.6	0.4	14.62**
*Undibacterium seohonense*	KC735151 (100)	2.6	2.4	0.2	11.97***
*Ideonella *sp.	JF176654 (100)	1.6	0.9	0.7	3.73
*Variovorax paradoxus*	MN684277 (100)	1.6	1.0	0.5	4.56
Uncultured *Rhodobacter *sp.	MN493576 (100)	1.6	0.3	1.3	5.27
*Pseudorhodoferax* sp.	FPLS01023064 (100)	1.1	1.0	0.1	4.89**
*Haliscomenobacter hydrossis*	NR_074420 (100)	0.9	0.3	0.6	2.88
*Pelomonas *sp.	KX508949 (100)	0.8	0.5	0.2	2.52
*Dongia *sp.	FPLS01061553 (100)	0.8	0.02	0.7	4.15
*Hydrogenophaga* sp.	HAFE01076884 (100)	0.5	0.2	0.4	1.79
*Methyloversatilis *sp.	FJ660513 (100)	0.5	0.1	0.4	1.58
*Paucibacter* sp.	FPLS01019111 (100)	0.4	0.2	0.2	1.33
*Candidatus Methylopumilus planktonicus*	FN668046 (100)	0.2	0.03	0.2	0.94
Uncultured *Myxococcales bacterium*	DQ646306 (97.9)	0.03	0.02	0.01	0.07

The respective contribution of these 16 OTUs to the distance-based distinction between treatment types was estimated by ‘simper’ analysis (1000 permutations), with asterisks denoting significance.

** *p*< 0.01; *** *p*< 0.001.

**Figure 1:**
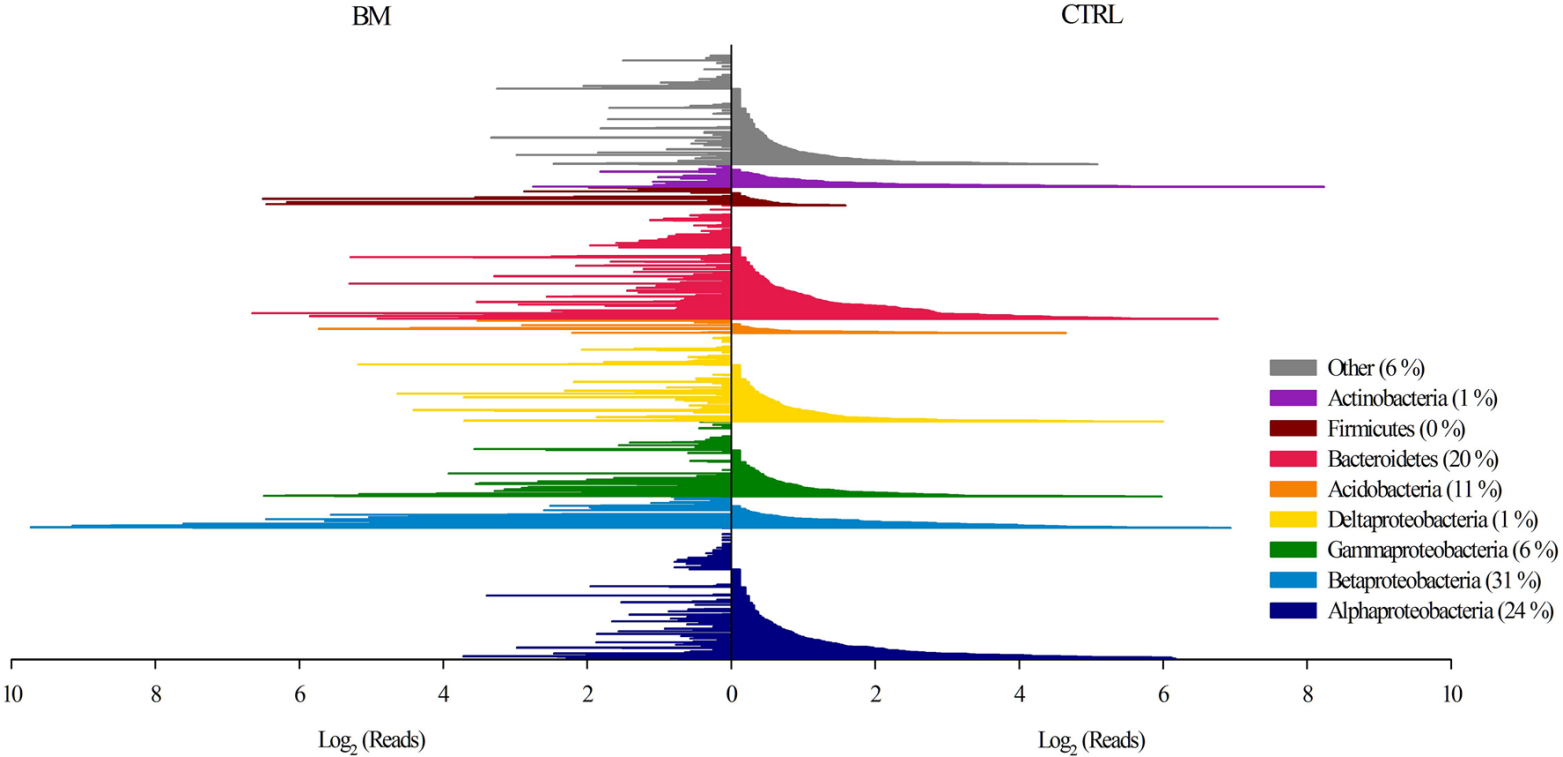
Distribution of phylogenetic groups in GDM biofilms with (BM) and without (CTRL) addition of cyanobacterial biomass. The right side of the graph plots OTUs in CTRL in ascending order of read numbers, and the left side the corresponding read numbers of the same OTUs in BM. Average read numbers (log_2_ transformed) of all 8 samples per treatment are depicted. A total of 63 OTUs significantly contributed to the distance-based separation of the two treatments (SIMPER analysis, *p* < 0.05). The distribution of these treatment-discriminating OTUs across the different phylogenetic groups is denoted in brackets after the name of the respective group.

In general, only very few OTUs on GDM biofilms were affiliated to the genotypes that are typically found in freshwater bacterioplankton^30^. Proteobacteria represented 51% of the total amount of OTUs in biofilm communities and 61% of the total amount of reads (Fig. 1). However, many OTUs within all phylogenetic groups of biofilm bacteria were treatment-specific, i.e., were either absent or had considerably lower read numbers in one of the treatments. A total of 63 OTUs significantly contributed to the distance-based separation of the BM and CTRL treatments (SIMPER analysis, *p* < 0.05). The numbers in brackets after the colour codes of the individual phylogenetic groups in Fig. 1 report their relative distribution. The majority of the treatment-discriminating OTUs were affiliated with Betaproteobacteria (31%), Alphaproteobacteria (24%), and Bacteroidetes (20%).

### Community structure and assembly processes

**Figure 2:**
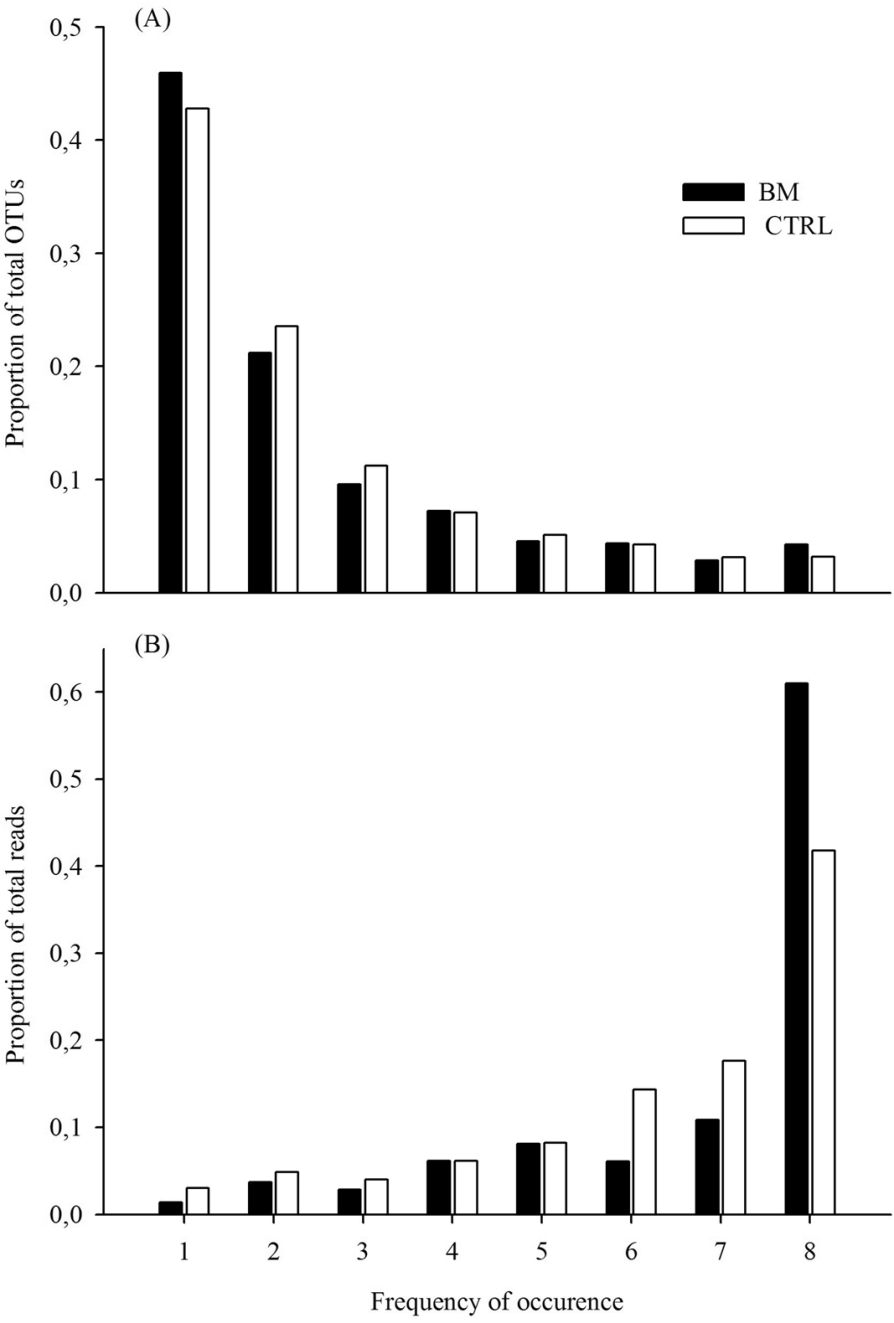
(**A**) Numbers of OTUs that were present in 1 to 8 biofilm communities of either treatment. (**B**) Summed read numbers of these OTUs. OTUs that occurred in all 8 communities of the CTRL treatment formed a significantly smaller proportions of total reads than in the BM treatment (Student’s *t* test, *p* < 0.05).

More than 40% of OTUs from either treatment only occurred in a single biofilm community (Fig. 2). By contrast, only 49 and 75 OTUs were present in all eight samples of the BM and CTRL treatments, respectively. The subset of ‘single sample’ OTUs had significantly more reads in the CTRL than in the BM treatments, whereas the opposite was the case for those OTUs that occurred in all communities of a treatment type (Student’s *t* tests, n = 16, *p* < 0.01).

**Figure 3:**
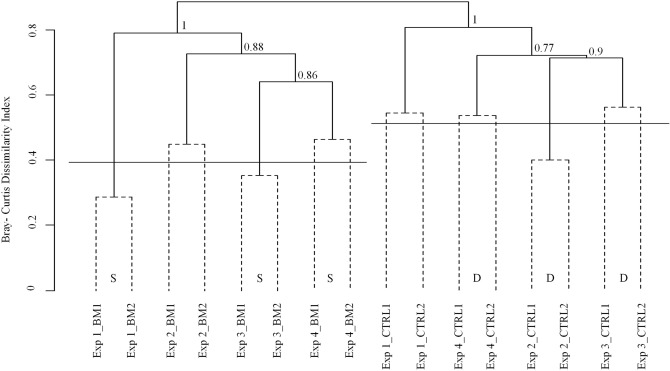
Average linkage clustering of all samples from the four experiments according to Bray–Curtis dissimilarity. The stability of the main branches was tested by bootstrapping (1,000 interactions). Similarity profile analyses (α = 0.001) identified 8 significantly different groups that corresponded to the individual experiments (solid lines). The horizontal lines are the average dissimilarity of biological replicates within individual experiments (BM: 39%; CTRL: 51%), which is significantly smaller in the BM treatment (1-sided *t* test, *p* < 0.05). S, D: experimental replicates that were significantly more similar or dissimilar than expected by chance (modified Raup-Crick index > 0.95 or < − 0.95, respectively).

Average linkage clustering of Bray–Curtis dissimilarities indicated a general separation between treatment types (Fig. 3), i.e., the communities of the BM treatments from all 4 experiments were more similar to each other than to the corresponding samples from the CTRL treatment. Similarity profile analysis showed that all phylogenetic groups except Firmicutes significantly contributed to this separation, albeit to a variable extent (Fig. 1). The clustering of only those OTUs that occurred in all biofilms (Table 1) also resulted in the perfect separation of samples according to treatment type (Bray–Curtis dissimilarity: 78%, data not shown). Five of these 16 ‘universal colonizers’ significantly contributed to this separation, all of them with higher read numbers in the BM treatment (Table 1).

The separation of communities according to treatment type was further corroborated by an error-free classification by Random Forest (RF) analysis (Out-of-Bag Error (OOB) = 0%, number of trees = 1,000, number of samples = 16, categories = 2). However, a RF classification of samples according to experiment was also remarkably accurate, and only 1 out of 16 samples was misclassified (OOB = 6.25%, number of trees = 1,000, number of samples = 16, categories = 4). The 20 OTUs that were most relevant for classification according to treatment type and experiment are listed in suppl. Tables S1 and S2, respectively.

Biological replicates of the BM treatments had significantly lower beta diversity (dissimilarity values: 0.39 ± 0.07) than those of the CTRL treatment (0.51 ± 0.07; one-tailed Student’s t-test, n = 8, *p* < 0.05) (Fig. 3). Moreover, Raup-Crick indices showed that the biological replicates of the BM treatments were significantly more similar to each other than by chance (RC > 0.95) in 3 out of 4 experiments, whereas the opposite was the case for replicates of the CTRL treatments (RC < -0.95).

**Figure 4:**
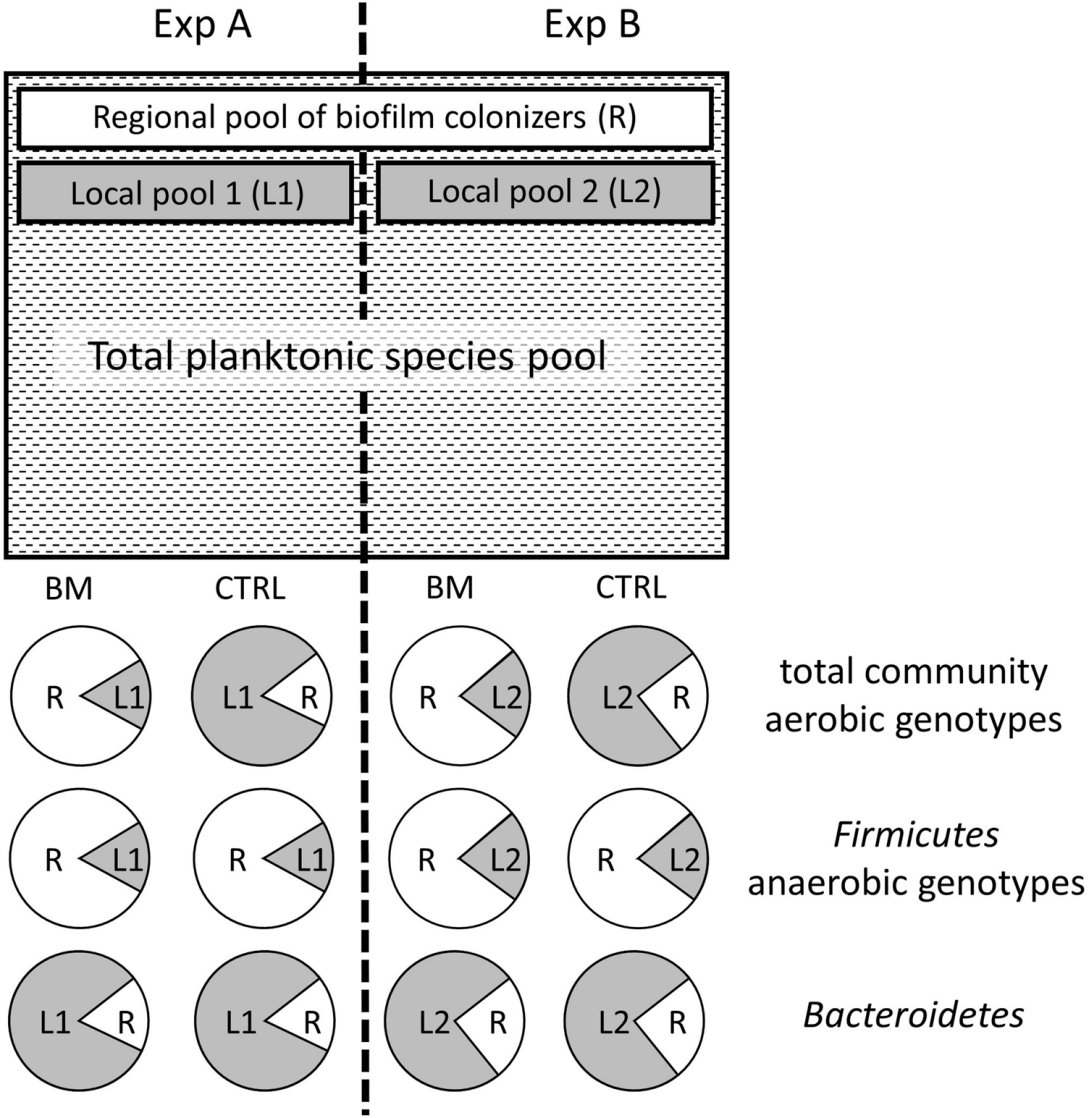
Conceptual depiction of biofilm community assembly in individual GDM experiments with (BM) and without (CTRL) addition of cyanobacterial biomass, as deduced by null model analysis (Normalized Stochasticity Ratio). The ‘regional pool’ of biofilm colonizers is defined as those species that were present in the feed water of all experiments, whereas the ‘local pool’ refers to genotypes that were specific to the feed water of a single experiment.

Null model analysis of community assembly processes indicated that the biofilms of the CTRL treatments were predominantly shaped by the ‘local’ pool of OTUs from the corresponding experiments (NST: 26%). By contrast, a significantly higher (*p* < 0.001) influence of the ‘regional’ pool of OTUs (i.e., that were present in several or all experiments) was found for biofilms of the BM treatments (NST: 66%). A graphic interpretation of these results is given in Fig. 4. In addition, assembly processes within the two treatments were also assessed for each of the phylogenetic lineages depicted in Fig. 1. Striking differences in treatment-specific NST values to those of the total community were observed for some phylogenetic groups, most prominently for Bacteroidetes and Firmicutes (Fig. 4, suppl. Table S3).

### Aerobic and anaerobic subcommunities

39% of all OTUs representing 78% of total reads could be assigned to either aerobic or anaerobic taxa, based on information on their closest cultured relatives. If possible, anaerobic taxa were further split into strict (272 OTUs) and facultative (62 OTUs) types. However, since both groups behaved similarly in the below described analyses, this distinction was abandoned for the sake of increased statistical power.

Both, the aerobic and anaerobic sub-communities were clearly distinct between treatments, as reflected by average linkage clustering of Bray–Curtis distances (data not shown) and highly accurate RF classification (OOB_aerobic_ = 0%; OOB_anaerobic_ = 6.25%; number of trees = 1,000, number of samples = 16, categories = 2). Null model analysis performed on the sub-community of aerobic genotypes yielded similar results to the complete dataset, i.e., a significantly higher impact of OTUs from the local pool on the CTRL (NST: 26%) than on the BM (NST: 65%) biofilm communities (*p* < 0.005). By contrast, the sub-communities of anaerobic genotypes were always predominantly recruited from the regional OTU pool irrespective of treatment type (NST_CTRL_: 64%; NST_BM_: 91%; *p* > 0.05) (Fig. 4). This conclusion was further supported by the poor RF classification of anaerobic genotypes according to experiment (OOB = 44%; number of trees = 50,000, number of samples = 16, categories = 4).

## Discussion

It is still challenging to accurately quantify the ‘rare biosphere’ of potential biofilm colonizers in the bacterioplankton^31,32^ and, thus, to study biofilm assembly processes by direct comparison with the source community. However, other approaches help to identify factors that modulate the respective importance of species sorting, immigration and stochasticity in biofilms, e.g., the analysis of communities in the context of the biotic and abiotic variation of the surrounding water^33,34^, or in their response to habitat manipulation^35^.

The diversity (richness) of the GDM biofilm communities was significantly lower in the substrate amended BM treatments than in the CTRL. This is the opposite of what has been described for planktonic diversity in oligotrophic ponds^36^, or groundwater microbial assemblages^35^, but agrees with observations on microbial assemblages in eutrophied lake sediments or from a marine phytoplankton bloom^37,38^. Such seemingly contradictory findings might potentially be reconciled by assuming a hump-shaped relationship between microbial diversity and productivity, as observed in soil^39^: On the one hand, severe substrate limitation will impose restrictions on the survival of many genotypes and select for a small set of oligocarbophilic specialists. On the other hand, extremely high substrate levels will specifically favour the most rapidly growing ‘opportunistic’ genotypes, which in turn might negatively affect the growth of others by releasing waste products or toxins.

BM treatments also featured a significantly higher NST score than the CTRL. At a first glance, it appears paradoxical that these communities should be mainly shaped by neutral processes such as immigration and ecological drift at arguably more selective conditions. However, null-model-based approaches are very sensitive to regional pool pre-selection: Biofilm communities consist of a subset of the bacterioplankton source assemblage that is already strongly filtered by deterministic processes^4^. Our analysis thus assessed if the individual communities tended to be formed by bacteria that were able to colonize the biofilms and at the same time were specific to individual experiments (‘deterministic’ with respect to the impact of the local species pool) or by such genotypes that were present in all source assemblages (‘stochastic’, i.e., mainly formed by the regional species pool) (Fig. 4). Thus, the high NST value of the BM treatments indicates that colonization of these biofilms was largely unrelated to priority effects, the compositional variability or the species turnover in the source water between experiments, and mainly driven by niche-related processes. The importance of environmental filtering in BM treatments is also suggested by the significantly higher similarity of the biological replicates within single experiment, i.e., lower Bray–Curtis distances (Fig. 3) and high Raup-Crick indices. Our findings agree with observations from parallel bioreactors, where higher substrate supply led to the formation of less divergent communities^40^.

Competitive exclusion might be directly related to the higher availability of organic C and nutrients, e.g., by favouring more opportunistically growing genotypes^41^. Eutrophication and high organic load increased the importance of niche-driven selection in coastal bacterioplankton assemblages^42^. However, two indirect effects of biomass addition also need to be considered: For one, more extreme habitat conditions, in particular the more pronounced oxygen limitation due to higher respiration^43,44^, might also have favoured niche-driven assembly processes in BM treatments. Environmental filtering was most prominent at conditions that most strongly deviated from those of the source assemblages (i.e., draught) in the sediments of a salt pan^45^. Secondly, due to the added biomass the transmembrane flux in the BM treatments was on average between 2–3 times lower than in the CTRL treatments^27–29,43^. Thus, the observed assembly patterns might at least in parts be due to ‘mass effects’, i.e., the successful colonizers of BM biofilms from the regional species pool (several of which are listed in Table 1) might have simply been more abundant in the source water than their competitors from the local pool.

The respective importance of assembly processes such as immigration and local selection is usually assessed at the level of entire microbial communities, implicitly assuming that these processes indiscriminately act on different community components. This has, e.g., been challenged by a separate assessment of the selection forces acting on common vs. rare members of microbial assemblages^31,32,46^. Biofilms are spatially heterogeneous habitats, featuring a complex three-dimensional architecture with pronounced horizontal and vertical gradients of oxygen or substrates^43,47^. It is conceivable that there might be differences in the assembly processes of distinct subcommunities that are either limited to particular microniches, such as obligate anaerobic bacteria^48^, or that share fundamental life style related traits^49^.

We found that niche-driven and neutral processes may selectively act on different compartments of GDM biofilm assemblages: In contrast to their aerobic counterparts, OTUs with an anaerobic metabolism were not selected from the local (i.e., experiment-specific) species pool in the CTRL treatment. Following the argument made above, we conclude that their establishment in GDM biofilms was thus mainly governed by niche-driven processes. Moreover, the clear difference in the composition of the anaerobic subcommunities between the two treatments suggests that anaerobic genotypes originated from several sources with different habitat properties, e.g. from sinking ‘lake snow’ particles^50^ vs. from food pellets within zooplankton carcasses^51^. The contrasting availability of organic C in the two treatments might have been a direct selective factor, yet it probably also shaped biofilm communities indirectly by defining the spatial patterns of oxygen distribution: On the one hand, interspersed anoxic microniches within a largely oxygenated matrix would lead to high habitat heterogeneity and variable selection in the CTRL biofilms^44^. This agrees with the more than twice as high number of anaerobic OTUs that were exclusive to this treatment (159 vs. 70 OTUs). Moreover, 98% of the 605 reads affiliated to the *Nitrosomonadaceae* (microaerophilic lithoautotrophic ammonia oxidizers) originated from the CTRL treatment, suggesting an interplay of oxygen gradients and C limitation. On the other hand, the high loads of organic material in the BM treatments likely led to considerable lower redox conditions in large areas of these biofilms. This conclusion is supported by the observation that > 90% of reads from OTUs affiliated with sulphate reducing bacteria (total: 1536 reads) and with the strictly anaerobic *Clostridiales* (total: 6,413 reads) were found in this treatment. BM biofilms also featured tenfold higher read numbers of the most common ‘globally occurring’ OTU (Table 1) that is closely related to the facultative iron reducer *Rhodoferax ferrireducens* (NR_074760, 98.8% sequence identity). In addition, there was evidence that microbes from individual phylogenetic groups did not adhere to the overall pattern of community assembly (Suppl. Table S3). In particular, OTUs affiliated with Bacteroidetes always tended to be recruited from the local species pool irrespective of treatment type, whereas the opposite was the case for Firmicutes (mainly consisting of Clostridiales classified as anaerobes). It has been argued that higher bacterial taxonomic ranks may to some extent also be ecologically meaningful units^52^, possibly because complex life style traits encoded by many genes are also phylogenetically conserved^49^.

The addition of cyanobacterial biomass led to a significant shift towards a small subset of ‘globally occurring’ treatment-specific colonizers of GDM biofilms (Fig. 2) both, within and between experiments. Moreover, the treatment types could be unambiguously separated by only considering 5 ubiquitous genotypes that all had 5 to 10 times higher read numbers in the BM treatment (Table 1). Our observation of a few ‘universally occurring’ indicator species for the addition of cyanobacterial biomass to GDM biofilms is derived from experiments conducted in 2011, 2014 and 2017, and thus appears to be rather robust with respect to variability of the source assemblages. Moreover, all 5 of the above mentioned OTUs were also present in biomass-amended GDM biofilms fed with water from a stream^28^. Two of them, *Rhodoferax* sp (LN870966) and *Undibacterium seohonense* (KC735151), also proliferated in GDM biofilms after addition of starch^43^, suggesting that their role as indicators might go beyond a particular lake and carbon source. Metabarcoding of pro- and eukaryotic marker genes from different habitats -including biofilms^53^- is increasingly regarded as a powerful tool for future environmental monitoring^54,55^. Recently, Keeley et al. reported that a small number of abundant microbial genotypes within operationally defined ‘eco-groups’ could accurately predict the enrichment of benthic habitats in salmon farms^56^. It is conceivable that bacterial biofilms in various technical or natural systems might also feature a small set of omnipresent reliable meters for fundamental aspects of habitat conditions such as substrate levels. Pronounced cyanobacterial blooms are a frequent phenomenon in many eutrophied lacustrine systems, resulting in concentrations of particulate organic carbon that are in the range of our experimental addition in the BM treatments^57^. Our study might thus help to develop a better understanding of the microbial communities that develop on ultrafiltration membranes or in sand filters fed with water from such systems.

## Materials and methods

### Experimental systems

Microbial biofilms grew on the ultrafiltration membranes (150 kDa nominal cutoff, polyethersulfone membrane; Microdyn Nadir, Wiesbaden, Germany) of experimental gravity-driven water filtration (GDM) systems. Water from Lake Zurich, Switzerland (location: 47°19′13.24″ N, 8°33′11.86″ E) obtained from the aerobic layer at 5 m depth was used as continuous feed in four independent experiments conducted over a period of 6 years (Table 2)^27–29^. Lake water was collected in sedimentation tanks and the GDM systems were supplied from the overflow. More details and a graphic depiction of the experimental systems are given in Silva et al.^27^ and ^28^.

**Table 2:** Information on the 4 experiments

Experiment	Start date of experiment	Duration (days)	Dry cell weight of the added biomass (BM treatment) (mg day^−1^)
1. Kohler et al. ^29^	16.06.2011	21	14.8
2. Silva et al.^27^	04.06.2014	23	15.2
3. This study	16.10.2014	23	13.9
4. Silva et al.^27^	17.02.2017	30	17

Citations are given for published data sets.

In each experiment, two treatments were run in parallel with the same feed water: One set of GDMs (2–3 biological replicates per experiment) received a daily dose of destroyed biomass from an axenic culture of *Microcystis aeruginosa* PCC7806 (BM treatment, Table 2). Another set of GDMs was operated without additional manipulation (CTRL treatment) until biofilm maturation (2–3 weeks). The input of particulate organic carbon from lake water to the CTRL treatments was by approximately 2 orders of magnitude smaller than that of the BM treatments, as estimated from the concentrations of particulate organic matter (POC) in Lake Zurich^58^ and daily flux rates through GDM systems^27,28,43^.

### DNA extraction and sequencing

At the end of each experiment, the biofilms were collected and DNA was extracted using the DNeasy PowerBiofilm Kit (Qiagen, Germany) according to the manufacturer’s specification (but extending the removal step for inhibitors to one hour). The recovered DNA was dissolved in 10 mM Tris buffer and stored at -20 °C until further processing.

Partial 16S rRNA genes were amplified with a primer pair that excludes chloroplasts and cyanobacteria (799F-1115R)^59,60^, but using a modified reverse primer for increased coverage^27^. Sequences were obtained by Illumina MiSeq paired end (2 × 300 bp) sequencing. Sequencing data from two of the four experiments (Exp 2, 4) have been published before, albeit separately^27,28^. The stored DNA extracts from another published experiment (Exp 1)^29^ were reamplified and resequenced on the Illumina platform for the purpose of this study. Samples from all experiments were processed by the same company (LGC Genomics, Germany).

Amplicon sequence data from two biological replicates of each treatment type from all four experiments were collectively re-analysed in order to produce a single, coherent set of operational taxonomic units (OTUs). The raw sequence data processing, the definition of OTUs and their taxonomic assignment according to the SILVA taxonomy (version 132)^61^ were performed by an in-house pipeline as described previously^27^.

### Data treatment and statistical analysis

Data analysis was carried out in R^62^. Biofilm communities were clustered by the average linkage method based on their Bray–Curtis dissimilarity. This was done for the complete dataset and separately for each of the phylogenetic groups depicted in Fig. 1. Similarity profile analysis was used to establish significant clusters (1,000 simulations, α = 0.001) and the stability of the main branches was estimated by bootstrapping (1,000 bootstraps). These calculations were performed with the R packages *clustsig*, *vegan*, and *fpc*^63–65^. Similarity percentage (Simper) analysis based on Bray–Curtis dissimilarity (R package *vegan*) was used to examine which of the ‘core’ genotypes that were present in all samples (Table 2) significantly contributed to the separation between treatments.

Differences in various community parameters between the BM and CTRL treatments were tested for significance by Student’s t-tests, following a prior testing for normality (Shapiro–Wilk test) and homoscedasticity (Levene’s test) with the R package *car*^66–68^. Proportional data were logit transformed prior to statistical testing^69^. In order to account for a possible confounding effect of differences in alpha diversity between treatments^70^ we also calculated the pairwise Raup-Crick indices (RC) of the biological replicates using the R package *NST*^71^.

A Random Forest (RF) classification (R package *randomForest*) was performed to assess how accurately the samples could be assigned to treatments or experiments by means of their respective community composition, and to identify the responsible OTUs^72,73^. The RF algorithm is a supervised machine learning model based on decision trees to classify data into pre-defined categories. The number of required decision trees (range: 1,000 to 50,000) was set to a value where the out-of-bag (OOB) error was stable. The Gini Impurity Metric was used as criterion to identify OTUs that were most relevant for the classification.

The influence of the ‘local’ and ‘regional’ pools of OTUs (i.e., OTUs that tended to be more or less specific for a single experiment or treatment) on biofilm community assembly in the two treatments was estimated by a null model analysis using the Normalized Stochasticity Ratio (NST; R package *NST*)^71^. NST values > 50% generally point to a predominance of stochastic processes on community assembly^71^. In our specific context, high NST values speak for the importance of biofilm colonizers from the ‘regional pool’ of OTUs that were present in similar read numbers across several or all experiments. Differences between NST values were assessed for statistical significance by bootstrap analyses (1,000 bootstraps).

## Supplementary information

Supplementary file1.
