# Supplementary material for: Biomass addition alters community assembly in ultrafiltration membrane biofilms
Source: Sci Rep. 2020 Jul 14;10:11552. doi: 10.1038/s41598-020-68460-x (PMC7360762; doi:10.1038/s41598-020-68460-x)
Supplement: Supplementary file 1 — Supplementary file1. [file 41598_2020_68460_MOESM1_ESM.pdf]

Biomass addition alters community assembly in ultrafiltration membrane biofilms

Marisa O.D. Silva and Jakob Pernthaler*

Supplementary Material:

Table S1: OTUs that most substantially contributed to a correct Random Forest classification according to treatment type (as derived from the Gini impurity metric). The frequency of occurrence in each treatment is denoted between brackets in the two rightmost columns of the table.

**Taxonomy**	**Accession number (% identity with closest known sequence)**	**Mean Decrease Gini**	**BM treatment: % of reads (number of communities)**	**CTRL treatment: % of reads (number of communities)**
Candidatus Methylopumilus planktonicus	FN668046 (100)	0.134	0.03 (8)	0.2 (8)
Uncultured *Nanopelagicales* (acI)	HQ214963 (100)	0.120	-	0.2 (8)
Woodsholea maritima	FM886859 (100)	0.105	0.01 (5)	0.4 (8)
Uncultured *Gamma-proteobacterium*	HQ010144 (97.9)	0.105	-	0.02 (8)
Uncultured *Aquimonas* sp.	KT308385 (99.7)	0.101	0.001 (2)	0.4 (8)
Uncultured *Chloroflexi bacterium*	JN409115 (90.9)	0.096	0.003 (4)	0.05 (8)
Sphingopyxis sp.	JF219913 (100)	0.096	0.05 (6)	1.2 (8)
Mesorhizobium sp.	KY445634 (100)	0.096	0.0005 (1)	0.03 (8)
Pseudomonas sp.	LN875097 (100)	0.095	0.08 (8)	0.001 (3)
Bdellovibrio sp.	AY168736 (100)	0.093	0.1 (8)	0.003 (2)
Candidatus Planktophila lacus	CP016780 (100)	0.090	0.001 (3)	0.3 (8)
Alpha proteobacterium	LC270264 (100)	0.087	-	0.08 (8)
Dongia sp.	HM921149 (100)	0.087	0.001 (2)	0.09 (8)
Candidatus Planktophila vernalis	FN668227 (100)	0.083	0.001 (1)	0.3 (8)
Undibacterium seohonense	KC735151 (100)	0.075	2.5 (8)	0.2 (8)
Nitrospira sp.	LN870809	0.071	0.0005	0.1

	(100)		(1)	(8)
Bradyrhizobium sp.	JF429058 (100)	0.071	-	0.04 (8)
Uncultured *Deltaproteobacterium*	AY689551 (99.3)	0.069	0.003 (2)	0.3 (8)
Reyranella sp.	MH669533 (98.6)	0.068	0.004 (4)	0.2 (8)
Fusibacter sp.	KM410449 (100)	0.064	0.80 (8)	0.01 (4)

Table S2: OTUs that most substantially contributed to a correct Random Forest classification of individual experiments (as derived from the Gini impurity metric). The frequency of occurrence in each experiment is denoted between brackets in the four rightmost columns of the table.

Taxonomy	Accession number (% distance to the closest cultured relative)	Mean Decrease Gini	Exp 1: % of reads (number of communities)	Exp 2: % of reads (number of communities)	Exp 3: % of reads (number of communities)	Exp 4: % of reads (number of communities)
Xanthomonadaceae bacterium	GQ902861 (93.36)	0.090	-	0.01 (4)	0.21 (4)	0.02 (4)
Pseudoxanthomonas sp.	LN560679 (100)	0.084	0.002 (4)	0.05 (4)	1.4 (4)	1.4 (4)
Bdellovibrio sp.	AY094107 (90.66)	0.078	0.01 (4)	-	0.002 (4)	0.001 (2)
Aquabacterium sp.	JF429319 (100)	0.076	0.001 (2)	0.05 (4)	0.03 (4)	0.1 (4)
Arenimonas sp.	JF429389 (100)	0.076	-	0.03 (4)	0.02 (4)	0.2 (4)
Rhodobacter sp.	FN668055 (100)	0.075	0.01 (3)	0.02 (4)	0.04 (4)	0.7 (4)
Azospirillum picis	MF062666 (100)	0.074	2.3 (4)	0.001 (2)	0.01 (4)	0.003 (3)
Uncultured *Sphingobacteriales bacterium*	JN679200 (99.65)	0.074	0.0005 (1)	0.04 (4)	0.5 (4)	0.04 (4)
Uncultured *Candidatus Gracilibacteria bacterium*	FJ902111 (94.39)	0.071	-	-	0.002 (4)	-
Azospirillum sp.	KC001708 (100)	0.070	0.02 (4)	-	-	-
Uncultured *Magnetospririllum* sp.	KX774239 (96.14)	0.070	-	-	0.001 (2)	0.2 (4)
Luteimonas sp.	LN566180 (100)	0.070	0.01 (2)	0.02 (4)	0.3 (4)	2.4 (4)
Lysobacter sp.	KX014807 (97.89)	0.069	-	0.001 (3)	0.5 (4)	0.01 (4)
Uncultured *Fibrobacteres bacterium*	KR813916 (89.97)	0.068	-	-	-	0.02 (4)
Sphingomonas sp.	HE965746 (100)	0.068	0.03 (4)	-	-	-
Sphingobacteriales bacterium	MN067550 (98.60)	0.067	0.02 (4)	0.02 (4)	0.1 (4)	0.01 (3)
Tahibacter sp.	JF176542 (100)	0.067	0.03 (3)	0.07 (4)	0.7 (4)	0.2 (4)
Pseudoxanthomonas sp.	FPLP01000084 (100)	0.066	0.002 (2)	0.01 (4)	0.3 (4)	0.4 (4)
Legionella waltersii	LT906442 (100)	0.066	-	0.02 (4)	0.02 (4)	0.02 (4)
Dinghuibacter sp.	KF827177 (100)	0.065	-	0.08 (4)	0.2 (4)	0.0005 (1)

Table S3: Treatment specific Normalized Stochasticity Ratio (NST) calculated separately for all phylogenetic groups depicted in Figure 1.

**Phylogenetic groups**	**NST (%)**	
	**CTRL**	**BM**
Alphaproteobacteria	45	72
Betaproteobacteria	49	67
Gammaproteobacteria	41	77
Deltaproteobacteria	70	56
Acidobacteria	62	69
Bacteroidetes	34	41
Firmicutes	70	82
Actinobacteria	78	67
